# ipaPy2: Integrated Probabilistic Annotation (IPA) 2.0—an improved Bayesian-based method for the annotation of LC–MS/MS untargeted metabolomics data

**DOI:** 10.1093/bioinformatics/btad455

**Published:** 2023-07-25

**Authors:** Francesco Del Carratore, William Eagles, Juraj Borka, Rainer Breitling

**Affiliations:** Manchester Institute of Biotechnology, Faculty of Science and Engineering, University of Manchester, Manchester M1 7DN, United Kingdom; Department of Biochemistry and Systems Biology, Institute of Integrative, Systems and Molecular Biology, University of Liverpool, Liverpool L69 3BX, United Kingdom; Manchester Institute of Biotechnology, Faculty of Science and Engineering, University of Manchester, Manchester M1 7DN, United Kingdom; Manchester Institute of Biotechnology, Faculty of Science and Engineering, University of Manchester, Manchester M1 7DN, United Kingdom; Manchester Institute of Biotechnology, Faculty of Science and Engineering, University of Manchester, Manchester M1 7DN, United Kingdom

## Abstract

**Summary:**

The Integrated Probabilistic Annotation (IPA) is an automated annotation method for LC–MS-based untargeted metabolomics experiments that provides statistically rigorous estimates of the probabilities associated with each annotation. Here, we introduce ipaPy2, a substantially improved and completely refactored Python implementation of the IPA method. The revised method is now able to integrate tandem MS fragmentation data, which increases the accuracy of the identifications. Moreover, ipaPy2 provides a much more user-friendly interface, and isotope peaks are no longer treated as individual features but integrated into isotope fingerprints, greatly speeding up the calculations. The method has also been fully integrated with the mzMatch pipeline, so that the results of the annotation can be explored through the newly developed PeakMLViewerPy tool available at https://github.com/UoMMIB/PeakMLViewerPy.

**Availability and implementation:**

The source code, extensive documentation, and tutorials are freely available on GitHub at https://github.com/francescodc87/ipaPy2

## 1 Introduction

LC–MS-based untargeted metabolomics is a key technology in systems biology (Nash and Dunn 2019). A single experiment measures thousands of mass spectrometry features, and their association with specific metabolites (i.e. their annotation) still represents a major challenge (Nash and Dunn 2019). Annotation often relies exclusively on the comparison of detected features with information reported in public databases. However, all features detected can be viewed as parts of a network, where the nodes (i.e. detected features) are connected by informative relationships (e.g. biochemical transformations, adduct formation, and isotopic connections). Considering this information, metabolite identification accuracy improves drastically, and several successful efforts in this direction have been reported (Chen *et al.* 2021, Zhou *et al.* 2022, Amara *et al.* 2022). One of the earliest of these tools is the Integrated Probabilistic Annotation (IPA) method (Del Carratore *et al.* 2019). IPA applies a Bayesian-based approach to incorporate different sources of information in the annotation process, including isotope patterns, adduct formation, and biochemical connections. It provides a statistically rigorous estimation of the confidence in each proposed annotation. Here, we report on a thoroughly revised and improved version of the IPA tool. The original IPA package was implemented as an R package, while the new implementation has been completely refactored in Python to improve the library functionalities and to facilitate future improvements and maintenance. Moreover, moving to Python greatly facilitates the integration of the IPA tool with the mzMatch pipeline and the newly developed PeakMLViewerPy visualization tool (https://github.com/UoMMIB/PeakMLViewerPy). Together with a user-friendly interface, ipaPy2 offers several improvements compared to its predecessor as described below.

## 2 Database and data preparation

Together with the library, a database containing the compounds present in the KEGG database (Kanehisa *et al.* 2017), the Natural Product Atlas (van Santen *et al.* 2022), and the compounds from the MoNa database (MoNA 2021) having at least one fragmentation spectrum acquired with a QExactive mass spectrometer is provided. To fully exploit the IPA method, users are strongly advised to build their own *ad hoc* database and to constantly update it. By updating the database with information about common adducts and in-source fragments, likely retention time ranges and fragmentation spectra, the IPA method integrates knowledge gained from previous experiments and iteratively improves the annotation process. The IPA method relies on the accuracy and completeness of the information stored in its database. When updating the database, it is important to be careful to avoid unwanted biases. For example, if the user assumes that all stereoisomers of the same compound elute at similar retention times, the same retention time range should be added for them. This will avoid biases toward specific stereoisomers. Both the database and the input datasets must adhere to a specific format that is detailed in the documentation (https://bit.ly/ipaPy2README). To this end, the data originating from an LC–MS-based untargeted metabolomics experiment need to be further processed. For example, it is necessary to cluster the features likely to be generated by the same metabolite, based on correlation across samples and retention time (RT), and to map the isotopes. This can be achieved either through functions provided within the ipaPy2 library or through widely used data processing software (Smith *et al.* 2006, Scheltema *et al.* 2011, Kuhl *et al.* 2012). Moreover, the tandem mass spectrometry (MS^2^) fragmentation spectra acquired must be assigned to the corresponding MS^1^ features upstream of the annotation pipeline, as detailed in the Data preparation section of the documentation (https://bit.ly/ipaPy2README).

## 3 IPA method

**Figure 1.**
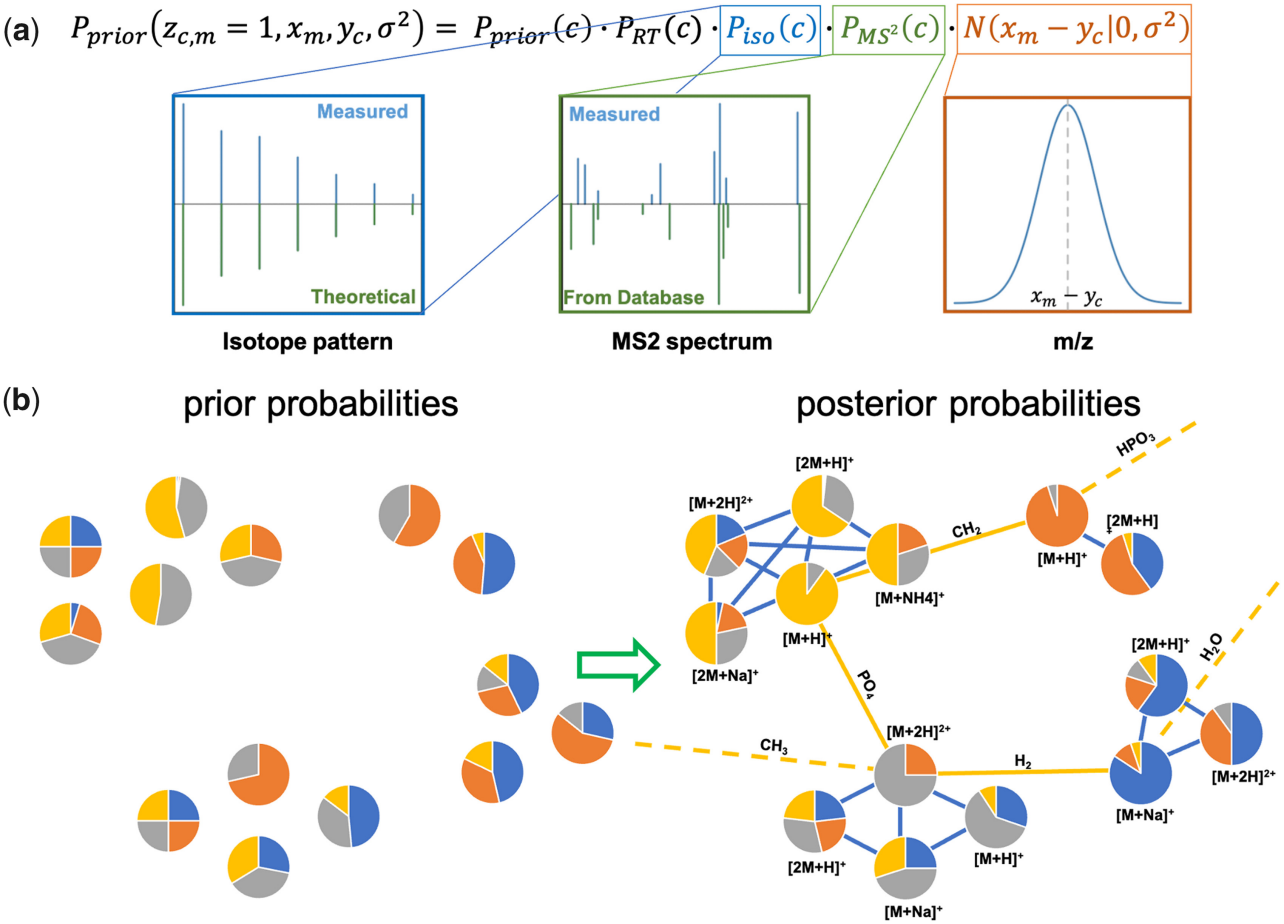
Overview of the IPA method. (a) Summary of the factors considered when evaluating the prior probabilities: prior belief, RT, isotope pattern, fragmentation spectra (MS^2^), and mass-to-charge ratio (*m*/*z*). (b) Graphical example of how the Gibbs sampler can evaluate the posterior probabilities by considering all possible biochemical connections and the connections between adducts and in-source fragments. Pie charts represent the probability assigned to each putative annotation.

A formal and extensive description of how the Bayesian statistics underlying the IPA method work is already available (Del Carratore *et al.* 2019). As summarized in Fig. 1, the IPA approach consists of two steps.

Step 1: Prior probabilities are evaluated considering: how likely it is for the compound considered being present in the sample; how close the measured RT is to what is reported in the database; how similar the MS^2^ spectrum (when acquired) is to what is reported in the database; and how close the measured mass-to-charge ratio is to the theoretical one (Fig. 1a). Isotope patterns are now considered as a unit, and their similarity to the theoretical isotope patterns is also included in the evaluation of prior probabilities.

Step 2: a Gibbs sampler is then used to estimate the posterior probabilities of all annotations by considering all possible biochemical connections and the connections between adducts and in-source fragments (Fig. 1b).

## 4 Isotope pattern as fingerprints

In the original implementation of the IPA method, isotopes were considered in the same way as any other feature, and the isotope pattern information was integrated in the annotation process within the Gibbs sampler. In the current implementation, isotopes are instead integrated into isotope fingerprints and used to compute the ‘isotope pattern score’ which is considered in the evaluation of the prior probabilities (step 1 of the IPA method). The Gibbs sampler is the most computationally demanding step in the whole annotation pipeline. The computation time of each iteration depends on the number of features and the number of hits in the database for each feature. As shown in Supplementary Fig. S1, the computational time for each iteration of the Gibbs sampler increases (in a non-linear fashion) with the number of features considered. Considering the isotope patterns fingerprints rather than single features drastically reduces the number of features in any dataset, therefore significantly reducing the computational time needed for the annotation. Upstream of the annotation process, it is therefore necessary to map the isotope patterns. This step can be performed through the ‘map_isotope_patterns()’ function provided within the ipaPy2 library or through other data processing software, such as mzMatch (Scheltema *et al.* 2011). For each feature with a detected isotope pattern, the theoretical isotope pattern associated with each possible annotation is calculated through the molmass python library (https://pypi.org/project/molmass/). The isotope pattern scores are then calculated by considering both the differences between theoretical and measured mass-to-charge ratios and the differences between measured and theoretical intensity ratios. The obtained scores are then used to update the probabilities associated with each annotation as described in Section 3.

## 5 MS^2^ data integration

Mass spectral fragmentation data are routinely acquired in untargeted metabolomics experiments and provide an invaluable source of information for the elucidation of chemical structures (Alseekh *et al.* 2021). The fact that such data were not considered in the original implementation of the IPA method represented one of its major limitations. The ipaPy2 package is now able to consider MS^2^ spectra when evaluating the probabilities associated with each possible annotation. For any feature associated with at least one measured MS^2^ spectra, this is achieved by comparing the measured spectra with all the spectra present in the database and associated with all the possible annotations. For each possible annotation, the highest cosine similarity score is considered the fragmentation pattern score. For all the annotations not having any fragmentation spectra stored in the database, a user-defined dummy fragmentation pattern score is used to allow a proper comparison. The resulting scores are then used to update the probabilities associated with each annotation as described in Section 3.

## 6 Example datasets

To demonstrate the applicability of the IPA method to different scenarios, the ipaPy2 library has been tested on four different datasets. The results of these analyses are available as extensively annotated Jupyter Notebooks, suitable for training purposes. The first dataset, introduced by Del Carratore *et al.* (2019), is a synthetic example where the features associated with 15 compounds were simulated (https://bit.ly/ipaPy2Synthetic). To demonstrate the increase in the annotation accuracy provided by the integration of fragmentation spectra in the annotation pipeline, MS^2^ data were also simulated. The second and third datasets were obtained from the analysis of different beer samples (https://bit.ly/ipaPy2Beer) and from the analysis of *Escherichia coli* extracts spiked with different concentrations of known standards (https://bit.ly/ipaPy2Ecoli), respectively. The fourth dataset, introduced by Ten-Doménech *et al.* (2020), was obtained from the analysis of human milk samples, where fragmentation spectra were acquired via a Data Dependent Acquisition strategy (https://bit.ly/ipaPy2Hmilk).

## 7 Integration with mzMatch

ipaPy2 was specifically designed to work with the mzMatch visualization tool and the .peakml data format (Scheltema *et al.* 2011). In fact, it includes a function able to extract the necessary data to run the IPA annotation from a .peakml file, as well as a function able to add the obtained annotations to the initial file. The resulting annotated file can then be easily explored via the newly developed PeakMLViewerPy visualizing tool (https://github.com/UoMMIB/PeakMLViewerPy). The integration of ipaPy2 with mzMatch is detailed in a Jupyter Notebook that can be found on Github at (https://bit.ly/ipaPy2mzMatch).

## 8 Conclusion

The ipaPy2 library represents a much improved and user-friendly implementation of the IPA method. In contrast to the previous implementation, ipaPy2 now considers isotope patterns as single units, which greatly reduces the computation time. Moreover, the accuracy of the method has been improved by considering MS^2^ spectra in the annotation process. ipaPy2 is designed to work with mzMatch and the .peakml data format, and the results can be easily accessed via the PeakMLViewerPy visualization tool.

## Supplementary Material

btad455_Supplementary_Data
