# Supplementary material for: ipaPy2: Integrated Probabilistic Annotation (IPA) 2.0—an improved Bayesian-based method for the annotation of LC–MS/MS untargeted metabolomics data
Source: Bioinformatics. 2023 Jul 25;39(7):btad455. doi: 10.1093/bioinformatics/btad455 (PMC10382385; doi:10.1093/bioinformatics/btad455)
Supplement: btad455_Supplementary_Data [file btad455_supplementary_data.docx]

*
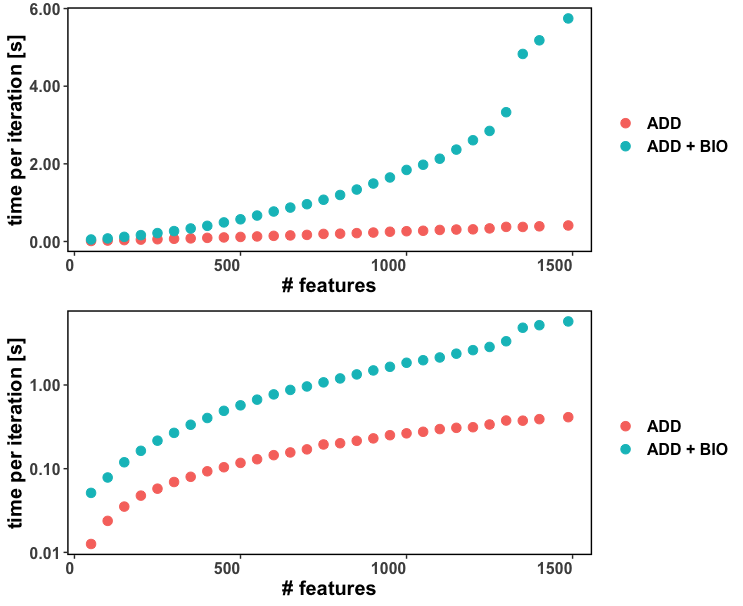
*

**Supplementary Figure 1** - The graphs show how the average time needed for each iteration of the Gibbs sampler increases with the number of features considered during the annotation process (considering only the adducts connections (red) or both biochemical and adducts connections (blue)) This analysis was performed using the “E. coli” example dataset by iteratively adding 50 features. The top graph is in linear scale, while the bottom graph is in log scale.
